# Phytochemical Profile, Bioactive Properties, and Se Speciation of Se-Biofortified Red Radish (*Raphanus sativus*), Green Pea (*Pisum sativum*), and Alfalfa (*Medicago sativa*) Microgreens

**DOI:** 10.1021/acs.jafc.3c08441

**Published:** 2024-02-23

**Authors:** Marilyn M. García-Tenesaca, Mercè Llugany, Roberto Boada, María-Jesús Sánchez-Martín, Manuel Valiente

**Affiliations:** † GTS Research Group, Department of Chemistry, Faculty of Science, Universitat Autònoma de Barcelona, 08193 Bellaterra, Spain; ‡ Plant Physiology Group (BABVE), Faculty of Biosciences, Universitat Autonòma de Barcelona, 08193 Bellaterra, Spain

**Keywords:** biofortification, bioactive compounds, microgreens, selenium, functional food

## Abstract

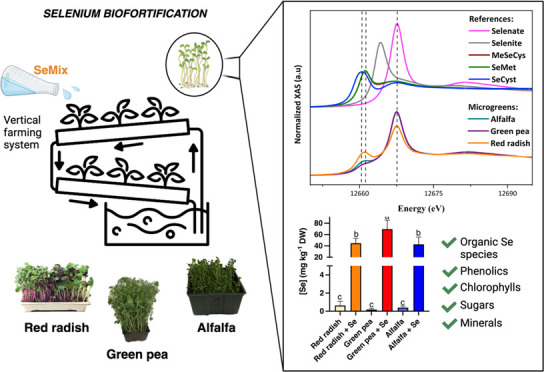

The impact of selenium (Se) enrichment on bioactive compounds and sugars and Se speciation was assessed on different microgreens (green pea, red radish, and alfalfa). Sodium selenite and sodium selenate at a total concentration of 20 μM (1:1) lead to a noticeable Se biofortification (40–90 mg Se kg^–1^ DW). In green pea and alfalfa, Se did not negatively impact phenolics and antioxidant capacity, while in red radish, a significant decrease was found. Regarding photosynthetic parameters, Se notably increased the level of chlorophylls and carotenoids in green pea, decreased chlorophyll levels in alfalfa, and had no effect on red radish. Se treatment significantly increased sugar levels in green pea and alfalfa but not in red radish. Red radish had the highest Se amino acid content (59%), followed by alfalfa (34%) and green pea (28%). These findings suggest that Se-biofortified microgreens have the potential as functional foods to improve Se intake in humans.

## 1 Introduction

Selenium (Se) is an essential microelement for humans and higher animals since it is the key component of biologically important selenoproteins. In humans, selenoproteins are involved in thyroid hormone metabolism, antioxidant defenses, and immune function. The recommended Se dietary intake for humans varies depending on age group and other factors (15–40 μg day^–1^ for ages from 1 to 13 years old, 55 μg day^–1^ from 14 to 50 years old, and 60–70 μg day^–1^ above 51 years old and for pregnant women or during breastfeeding).^1^ Unfortunately, around a billion people worldwide are affected by Se deficiency. The low Se status is associated with a wide range of pathological conditions such as Keshan disease, mood disorders, reduced male fertility, enhanced susceptibility to infections, and disturbance of thyroid function.^2,3^

Se biofortification techniques can directly provide plant-based Se-enriched food since plants are able to transform the less bioavailable inorganic Se species (selenite and selenate) present in soils to more bioavailable forms such as selenoamino acids (selenocystine (SeCyst), selenomethionine (SeMet), selenocysteine (SeCys), selenomethylcysteine (SeMeCys), γ-glutamyl-methylselenocysteine, dimethylselenide, and selenocystathionine), which are the desired Se forms for human diets. Therefore, this type of functional food is more convenient and economical than Se supplementation through the use of pills or capsules.^2^

In the last years, culinary herbs such as microgreens have become increasingly popular among consumers not only for their particular flavors, crunchy textures, and colors that make dishes more attractive but also for their high nutritional value.^4^ They are characterized by being rich in phenolic compounds, vitamins, antioxidants, and macro- and microelements at levels higher than seeds.^2,3,5^ These immature plants consist of cotyledons, stems, and a pair of true leaves, allowing them to be produced in a short time and in large quantities due to the less space required for their cultivation compared to adult crop plants.^6,7^

Phenolic compounds are a large group of secondary metabolites in plants that are related to defense responses.^8^ Moreover, these metabolites are well-known to show numerous bioactive properties, such as antioxidant and anti-inflammatory.^9^ Carotenoids and chlorophylls are the main pigments found in plants. The color of microgreens is one of the main traits that affects their acceptability by consumers; therefore, it is an important parameter defining their quality. It is also reported that carotenoids are bioactive compounds that have a great impact on human health by preventing several chronic diseases (diabetes, cancer, neurological disorders, immunity diseases, and others) and that are strongly related to the decrease of cardiovascular risk factors.^10^ Other compounds of interest are the plant sugars since these are considered a source of energy for the correct metabolism of the plant. After harvest, these molecules are crucial for keeping cells alive and ensuring a long shelf life of plant products.^11^

Alfalfa (*Medicago sativa*), green pea (*Pisum sativum*), and red radish (*Raphanus sativus*) microgreens are used as flavorful additions in some meals and sometimes, like other herbs, to replace salt, increasing the acceptance of these vegetables by the consumer.^4,12^ For that reason, they could be good candidates for biofortification with Se.

The Se biofortification of microgreens has only been studied in a few crop species^3,4,6^ and in some wild species.^13^ These studies have shown the positive effect of Se on different phytonutrients, such as phenolic compounds, mineral elements, pigments, vitamins, and the antioxidant status of the plant. Nevertheless, there is still limited information regarding all of these compounds. Widening our understanding of genotypic variation in the phytochemical composition and bioactive properties of microgreens can provide an important contribution to this growing industry as the relative abundance of bioactive compounds between species and their implications for their sensory and functional quality may support future species selection.

The main Se species found in most of the plants are the amino acids SeMet, SeCyst, and SeMeCys, together with the untransformed inorganic selenate and selenite.^2^ In green pea, red radish, and alfalfa, there are a few studies on the determination of Se species. Moreover, such studies have predominantly focused on the assessment of Se species during the adult stage of plant growth.^12^ Most speciation studies have been based primarily on high-performance liquid chromatography with inductively coupled plasma mass spectrometry (HPLC-ICP-MS). However, in certain cases, this approach has led to incomplete characterization of the overall species due to the low stability of specific Se species during sample pretreatment and incomplete recoveries using the methodologies employed. To overcome these limitations, direct speciation techniques, like X-ray absorption spectroscopy (XAS), offer a solution that allows speciation analysis of Se in solid form without the need for any extraction or pretreatment steps.^12,14^

Previous findings from wheat hydroponic cultures have shown that the application of a mixture of the two inorganic Se species (selenite and selenate) led to a more balanced distribution of Se through the plant with less toxicity compared to the effect of the application of each inorganic species independently.^15,16^ In this regard, the application of a mixture of the two Se species led to a level of C–Se–C organic compounds in wheat grains (62 ± 6%), which lies between those obtained for the inorganic treatments: selenate (74 ± 5% of C–Se–C) and selenite (57 ± 6% of C–Se–C). A similar effect was observed for C–Se–Se–C organic compounds since the Se mixture treatment yielded 38 ± 2%, whereas selenate and selenite produced 25 ± 2 and 44 ± 2%, respectively.^15^ Hence, comprehending the conversion of inorganic Se into organic species in microgreens is essential to enhance the efficiency of Se-biofortified foods while minimizing the potential risks of undesired toxicity. To date, there is a lack of reported data concerning the impact of biofortification with a mixture of selenate and selenite on the Se species accumulated in microgreens. The aim of this study is to evaluate the effect of this mixture of both Se species on the bioactive properties, compounds of interest, and elemental composition of microgreens and on the production of the Se organic species to obtain new plant products with high nutraceutical value. Additionally, we have analyzed the effect of Se on glucose and fructose contents. This is of industrial interest as it has a noticeable effect on the shelf life of the products; however, it has not yet been reported in any previous study. An improvement in the nutrient profile would allow these microgreens to be used as functional foods to improve human nutrition and tackle human Se deficiency.

## 2 Materials and Methods

### 2.1 Reagents

Folin–Ciocalteu reagent and nitric acid were purchased from VWR International (Barcelona, Spain); methanol, acetone, sodium carbonate, and Trolox standard were purchased from Fisher Scientific (Madrid, Spain); hydrogen peroxide was purchased from Panreac Applichem (Barcelona, Spain); gallic acid, diphenyl-2,4,6-trinitrophenyl iminoazanium (DPPH), sodium selenite, sodium selenate, seleno-l-methionine (≥98%), seleno-l-cystine (95%), and Se-(Methyl) selenocysteine hydrochloride (≥95%) were purchased from Sigma-Aldrich (St. Louis); and Milli-Q water was purified through a purification system from Millipore (Billerica, MA).

### 2.2 Plant Material and Growth Conditions

Red radish (*R. sativus* var. *vulcano*), green pea (*P. sativum* var. *balboa*), and alfalfa (*M. sativa* var. *victoria*) microgreens were cultivated by InstaGreen S.L. (Barcelona, Spain). Seeds were germinated and grown in cups (14 cm × 19 cm × 6 cm: *W* × *L* × *D*) using a cellulose substrate. These cups were laid on top of plastic trays mounted on a hydroponic vertical farming system in which the solution flows downward since the trays are slightly tilted. The dark period necessary during the germination process was 5 days for red radish, 8 days for green pea, and 4 days for alfalfa. The light exposures of the microgreens were 3, 6, and 9 days, respectively. Red radish and green pea were watered twice a day, and alfalfa was watered once a day for around 15 min. In all cases, only Se treatment was used for watering, and no additional nutrients were added. It was needed around 3 L of tap water with Se/day for the hydroponic vertical farming system. The light-emitting diode (LED) panel placed inside the vertical farming system guaranteed a homogeneous light distribution (81 μmol m^–2^ s^–1^) over the whole shelf surface. The culture conditions used were a light/dark photoperiod of 16/8h, a relative humidity of 58.6 ± 7.3%, and a temperature of 25.8 ± 0.6 °C. The nutrient solution had an electrical conductivity (EC) of 1.5 ± 0.3 mS cm^–1^ and a pH of 7.6 ± 0.2. All genotypes were harvested in the first stage of true leaf growth.

The Se treatment consisted of tap water with 20 μM Se based on a 1:1 molar mixture of sodium selenite and sodium selenate. This treatment was selected as the most suitable for this hydroponic cultivation following our previous findings^15,16^ and the results of other works in the literature^3,12,17^ in which similar concentrations did not induce any toxic effects to the plant. A control culture was obtained from a separated vertical farming system in which only tap water was used as the nutrient solution.

Samples were collected when the complete cotyledon and the first true leaf appeared, and the microgreens were cut just above the substrate level with sanitized scissors. Immediately after harvest, the fresh weight (FW) as g per cup was determined. Afterward, the microgreens were quickly frozen using liquid nitrogen and stored at −80 °C until lyophilization (Telstar Lyoquest, Spain). The dry weight (DW) as g per cup was measured after freeze-drying, and the dried seedlings were finely ground and stored at −20 °C until further analysis.

### 2.3 Elemental Analysis of Selenium and Minerals

Macro- (P, K, Mg, S, Ca) and micronutrients (B, Mn, Fe, Ni, Cu, Zn, Mo) were evaluated to assess the growth and development of crop plants due to their role in specific and essential physiological functions in plant metabolism.^18−20^ The elemental composition, including Se, was determined following the method described by Funes-Collado et al.^12^ with some modifications. Dried shoots (0.2 g) were microwave-digested (CEM Mars5 IP Microwave accelerated reaction system; Mathews NC) with 7 mL of ultrapure concentrated nitric acid (65% v/v) and 3 mL of hydrogen peroxide (30% v/v). The heating program for the digestion procedure was a 10 min ramp from room temperature to 90 °C; 5 min waiting at 90 °C; 10 min ramp from 90 to 120 °C; 10 min ramp from 120 to 180 °C; and 10 min waiting at 180 °C. After cooling, the digests were filtered using 0.22 μm syringe filters before further dilution and then analyzed by ICP-MS (XSeries 2, Thermo Scientific).

### 2.4 Determination of Chlorophylls (Chls) and Carotenoids (Car)

The freeze-dried plant material (0.1 g) was mixed with 10 mL of acetone/water (80:20, v/v) and stirred for 10 min. The mixture was centrifuged at 3500 ppm for 10 min, and the supernatant was filtered with a PVDF syringe filter of 0.45 μm. The filtered volume was made up of 25 mL with the solvent. The absorbance was measured at 440, 646, and 663 nm with a UV–vis spectrophotometer (Unicam UV-2 200, England). The concentrations of chlorophyll *a* (Chla), chlorophyll *b* (Chlb), and total carotenoids (Car) were determined using the following equations^21,22^
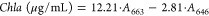
1
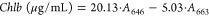
2
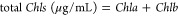
3
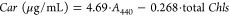
4where *A*_λ_ denotes the absorbance of samples at the corresponding wavelength (λ: 440, 646, and 663 nm).

### 2.5 Total Phenolic Compounds (TPCs) and Total Antioxidant Capacity (TAC)

To assess the influence of the Se biofortification on the bioactive properties of the microgreens, 0.5 g of fresh shoots was extracted with 5 mL of 80% methanol (v/v) for 2 h under continuous stirring in the dark. Then, the mixture was sonicated by using an ultrasonic bath (Bransonic 2510E-MT, Branson Ultrasonics Corporation, Danbury) at 100 W and 42 kHz for 15 min. The extract was centrifuged at 2200 rpm for 10 min, and the supernatant was filtered with a syringe filter of 0.45 μm. The supernatant was collected, and the pellet was resuspended in 5 mL of 80% methanol; the sonication and centrifugation steps were repeated once. Supernatants were combined to reach a final volume of 10 mL of extract and stored at −20 °C until analysis.

The Folin–Ciocalteu reagent method as described by Singleton et al.^23^ was used to determine the TPC. Briefly, 100 μL of methanolic extract was mixed with 0.5 mL of 0.2 N Folin–Ciocalteu reagent and 0.4 mL of 75 g L^–1^ sodium carbonate (Na_2_CO_3_). The mixture was vortexed and incubated at room temperature (20 °C) in the darkness for 2 h. Absorbance was measured with a plate reader spectrophotometer (Tecan Infinite 200 Pro, Austria) at 760 nm by using a 96-well plate. Gallic acid at 0–150 ppm concentrations was used as the standard. The results were expressed as milligrams of gallic acid equivalents (GAE) per g FW.

The total antioxidant capacity was determined by the DPPH method as described by Brand-Williams et al.^24^ The DPPH solution was prepared by dissolving di(phenyl)-(2,4,6-trinitrophenyl) iminoazanium in methanol to 0.1 mM concentration. 0.1 mL of methanolic extract of each sample or Trolox standard was mixed with 2.925 mL of DPPH solution, and after 30 min, the absorbance was measured at 515 nm with a plate reader spectrophotometer. Trolox equivalent antioxidant capacity (TEAC) results were expressed as μmol of Trolox equivalent per g FW.

### 2.6 Glucose and Fructose Quantifications

d-Glucose (Glu) and d-fructose (Fru) were determined by using an enzymatic kit assay (Biosystems, Spain). The d-glucose and d-fructose present in the sample can generate NADPH through the action of different enzymes, and the concentration of NADPH can be determined spectrophotometrically by monitoring the absorbance at 340 nm. Samples were pretreated before analysis by mixing 0.05 g of the lyophilized sample with 25 mL of Milli-Q water and heated to 60 °C for 5 min. They were then decolorized with 1:100 (g:mL) of poly(vinylpolypyrrolidone) (PVPP), mixed for 1 min, centrifuged at 3500 rpm for 10 min, and filtered with a syringe filter of 0.45 μm. The mixture with the working solutions of the kit was done according to kit instructions with 32 μL of sample. The results were expressed as milligrams of d-glucose or d-fructose per gram of FW based on the fresh weight mass obtained for each sample.

### 2.7 Se Speciation by X-ray Absorption Spectroscopy

X-ray absorption spectroscopy (XAS) offers the advantage of element-specific chemical speciation information without the necessity for any sample pretreatment. As a result, concerns about incomplete recoveries or the reactivity of the species are avoided. X-ray absorption near-edge structure (XANES) spectra were collected at Se K-edge at CLAESS beamline^25^ of ALBA synchrotron. The synchrotron radiation emitted by a wiggler source was monochromatized using a double-crystal Si(311) monochromator. The rejection of higher harmonics was done by choosing the proper angles and coatings of the collimating and focusing mirrors. Powdered microgreen samples (∼20 mg) were pressed into 5 mm pellets using a hydraulic press. Aqueous solutions of the Se references (sodium selenite, sodium selenate, seleno-l-methionine, seleno-l-cystine, and selenomethylcysteine) were measured in transmission mode (100–200 mM concentration) at room temperature using the in-house-designed 3D-printed liquid cell.^26^ The spectra of microgreens were collected in fluorescence mode using a multielement silicon drift detector with Xspress3 electronics, while the reference spectra were measured in transmission mode using gas ionization chambers. The spectra were collected on three spots for each sample to take into account possible inhomogeneities when mixing the powders of the replicates used. All of the measurements were performed at a liquid nitrogen temperature to diminish radiation damage effects. The data reduction, spectral normalization, and the linear combination fitting (LCF) analysis were performed using Athena software of the Demeter package.^27^ The goodness of fit was obtained by the *R*-factor (∑(data-fit)^2^/∑data^2^), which is a measure of the mean square sum of the misfit at each data point.

### 2.8 Statistical Analysis

The experiment involved 40 cups, two treatments, and three replicates per treatment and type of plant. The whole experiment was independently repeated twice under the same conditions to ensure reproducibility of the results. Data are reported as mean ± standard deviation (SD) of three measurements (except for fresh and dry weights and mineral analysis where five measurements were considered). A one-way analysis of variance (ANOVA) followed by Tukey’s test at a 0.05 probability level was performed for all variables. Principal component analysis (PCA) was performed to highlight correlations and visualize the effect of Se on the different parameters measured of each microgreen using PLS-Toolbox 4.0 with MATLAB software.

## 3 Results and Discussion

### 3.1 Plant Yield and Selenium Concentration

**Figure 1:**
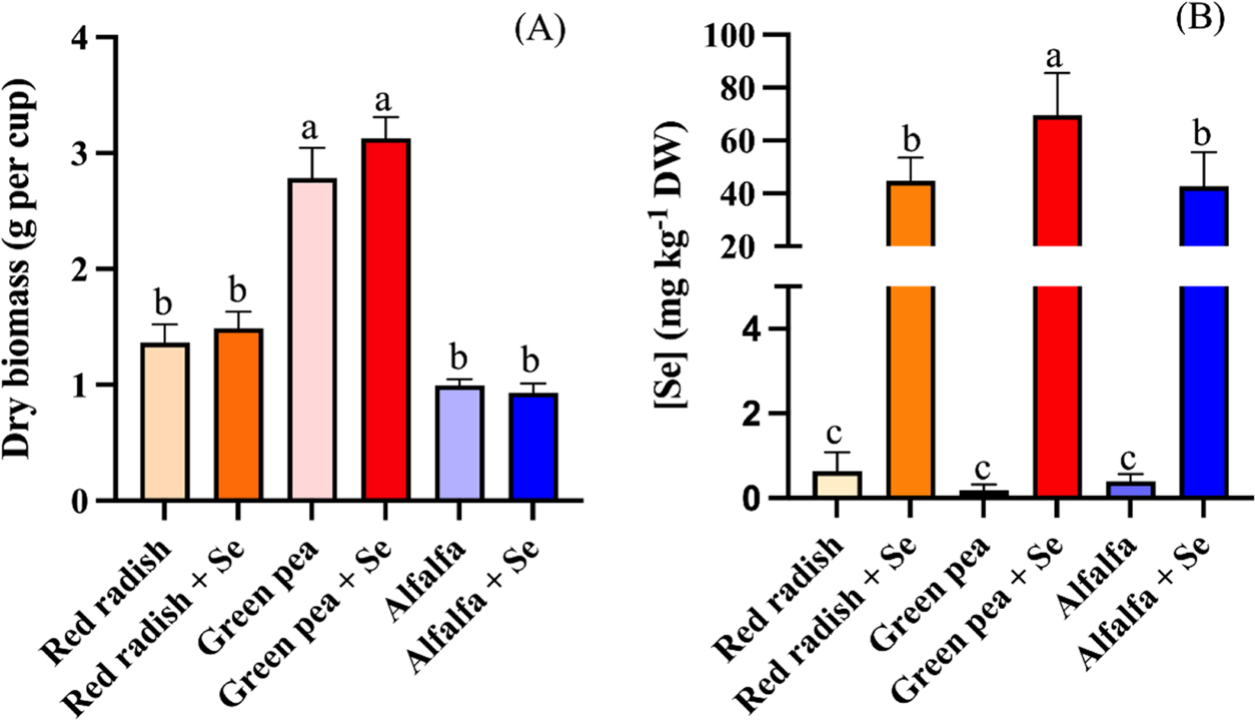
(A) Biomass expressed as the dry weight of red radish, green pea, and alfalfa microgreens treated with 20 μM selenium and their respective controls without treatment. (B) Total Se concentration in red radish, green pea, and alfalfa microgreens treated with Se and their respective controls. Bars indicate means (±SD; *n* = 5). Tukey’s significance at *p* ≤ 0.05 among treatments is indicated by different letters within the microgreens’ species.

The analysis of the DW of microgreens is reported in Figure 1A. Although the DW values of the Se-treated plants were slightly higher than those of the control for red radish and green pea, this variation is not statistically significant. The DW yield varied significantly among plant species, and it was not univocally correlated with growth time. The greatest biomass was obtained for green pea enriched with Se (3.1 ± 0.5 g DW per cup), and the lowest was alfalfa enriched with Se (0.9 ± 0.2 g DW per cup), which have a similar growing time. This work is in agreement with previous studies where similar concentrations of Se (13–32 μM) applied to different types of microgreens did not reduce the plant biomass.^3−5,12,28^ In addition, note that no toxicity effects were found on the different plant species throughout the growth period, such as chlorosis, dry leaves, growth retardation, or wilting.

The Se treatment applied significantly improved the amount of Se originally present in the microgreens. The highest Se concentration was detected for green pea (70 ± 16 mg·kg^–1^ DW), followed by red radish (45 ± 9 mg·kg^–1^ DW) and alfalfa (43 ± 13 mg·kg^–1^ DW) (Figure 1B). Our findings are in agreement with previous studies on microgreens of coriander, green basil, purple tatsoi, wheat, scallions, basil, cress, arugula, and mizuna,^3,4,6,28^ showing the efficacy of achieving biofortification of microgreens with Se. Experiments carried out by Funes-Collado et al.^12^ on alfalfa seedlings of similar growth stage demonstrated that applying ca. 25 μM of a mixture of selenate and selenite (1:1 molar ratio), the seedlings can reach Se concentrations of 132 mg Se kg^–1^ DW. In fact, it has been seen that Se uptake depends on the plant species, environmental conditions, and the time of exposure to it.^4,7^

So far, the literature does not provide enough consistent data regarding the amount of microgreens consumed daily per person to provide a meaningful average value. However, since microgreens are commonly eaten in small amounts, as they are used as a flavor enhancer or toppings in salads, meats, and soups, the average daily serving is expected to be around 10–20 g.^3,29^ An hydroponic study by Pannico et al.^3^ using 16 μM of Se in the form of sodium selenate to biofortify microgreens (coriander, green and purple basil, and tatsoi) reported Se levels in the range of 26–150 mg kg^–1^ DW. The authors suggested that, considering the consumption of a 10 g serving of fresh microgreens, these concentrations could be appropriated according to the recommended daily Se allowance (RDA) of 55 μg· day^–1^ in adults (70 kg body weight) and taking into consideration that less than 55 μg· day^–1^ results in a deficiency level and more than 400 μg day^–1^ of results in toxicity.^1,5^ In that sense, it could be said that the Se accumulated in the microgreens reported in our work is within the safe range of Se intake.

### 3.2 Influence of Se Biofortification on Mineral Elements

The effect of Se enrichment on the mineral element concentration showed differences, depending on the variety (Table 1). It is worth mentioning that microgreens are a good source of calcium (Ca) and potassium (K).^30^ In our study, the concentration of Ca in green pea and red radish was not significantly affected by Se biofortification; however, a significant decrease in Ca of 37% and magnesium (Mg) of 26% was observed in alfalfa biofortified with Se, compared with the control. Mezeyová et al.^28^ and Pannico et al.^3^ obtained similar results in some microgreens, where Ca was negatively affected by Se treatment. Longchamp et al.^31^ observed that in *Zea mays* tissues, the accumulation of Ca, Mg, Zn, Cu, and Fe is strongly related to the Se concentration and the inorganic form in which it was introduced into the nutrient solution. Ca plays a crucial role in osmoregulation and in maintaining the cation–anion balance, while Mg is essential for plant pigment synthesis and the activation of many enzymatic systems of photosynthesis and respiration.^31,32^ Our results indicate that the levels of both Ca and Mg were negatively affected by Se, implying a possible Se detoxification mechanism. This phenomenon is further related to a concurrent reduction in chlorophyll levels in alfalfa, as will be discussed later in the subsection describing the bioactive composition and antioxidant capacity (subseciton 3.3). In our study, K was not affected by Se treatment in alfalfa and green pea, but it was increased in red radish (∼21%). A similar increment of K (23%) was also observed in maize biofortified with 25 μM Se.^33^ Similarly, in green basil and coriander, an increase in K of 40 and 28%, respectively, was observed with an application of 16 μM Se, but an opposite effect was observed in tatsoi, decreasing this element by 30% when biofortifying only with 8 μM Se.^3^ Likewise, phosphorus (P), sulfur (S), and manganese (Mn) increased significantly around 13, 10, and 22%, respectively, in Se-treated red radish with respect to the control. Due to the chemical similarity between Se and S, Se is taken up, translocated, and assimilated through the S pathway. In the uptake of selenite, it is known that not only phosphate transporters are responsible for its assimilation, but it was also observed that aquaporins and silicon flux transporters are also involved in selenite assimilation, at least in rice.^34^ Therefore, it is evident that competition in the absorption of these elements can influence their accumulation in shoots. Moreover, the expression of sulfate transporters will vary depending on the level of S and the crop species.^35^ Previous reports have observed that Se can regulate Mn transport in plants.^34^ In Se-biofortified rice, it was observed that *OsNram5*, a member of the resistance-associated macrophage protein family and a pivotal transporter that regulates Mn uptake in plant shoots, was downregulated due to Se biofortification when selenite was used as Se treatment.^36^ While in *Brassica napus*, the expression of the *ZRT/IRT* family member (IRT1) was enhanced under selenite treatments, which positively mediated Mn translocation.^34,37^

**Table 1:** Macronutrient (Mg, P, S, K, Ca) and Micronutrient (Mn, B, Fe, Ni, Cu, Zn, Mo) Concentrations in Red Radish, Green Pea, and Alfalfa Microgreens Treated or Not with Se^a^

	macroelements (g kg^–1^ DW)				
	K	P	Ca	S	Mg
red radish	14 ± 0.9ac	11.2 ± 0.5a	7.4 ± 0.9a	20 ± 2a	4.9 ± 0.5a
red radish + Se	17 ± 2b	12.7 ± 0.9b	8.2 ± 1.2a	22 ± 2b	5.6 ± 0.9a
green pea	23.2 ± 0.9c	7.7 ± 0.4c	4.8 ± 0.5b	6.4 ± 0.2cd	3.1 ± 0.2b
green pea + Se	23 ± 2c	7.9 ± 0.3c	4.8 ± 0.8b	7.6 ± 0.6c	3.1 ± 0.3b
alfalfa	15 ± 1bc	6.2 ± 0.2d	6.8 ± 0.4a	5.2 ± 0.1d	4.2 ± 0.2a
alfalfa + Se	15 ± 2bc	6.3 ± 0.6d	4.3 ± 0.5b	4.8 ± 0.7d	3.1 ± 0.3b

	microelements (mg kg^–1^ DW)						
	Fe	B	Zn	Mn	Cu	Ni	Mo
red radish	63 ± 3a	36 ± 4ab	46 ± 4a	18 ± 2a	4.7 ± 0.8a	1.1 ± 0.1a	0.32 ± 0.06a
red radish + Se	71 ± 8ab	39 ± 6ab	52 ± 5a	22 ± 2bc	4.3 ± 0.3a	1.2 ± 0.3a	0.33 ± 0.06a
green pea	102 ± 11ab	39 ± 3ab	60 ± 4b	18 ± 2a	13.7 ± 0.2b	3.9 ± 0.7b	2.8 ± 0.6b
green pea + Se	109 ± 9b	34 ± 6b	60 ± 4b	18.5 ± 0.9a	14.1 ± 0.1b	5.1 ± 0.7c	2.6 ± 0.4b
alfalfa	545 ± 43c	42 ± 3a	45 ± 3ac	22.1 ± 0.6bc	13.3 ± 0.5b	4.6 ± 0.4bc	4.5 ± 0.2c
alfalfa + Se	542 ± 50c	36 ± 2ab	43 ± 2c	19.8 ± 0.6ac	13.1 ± 0.8b	4.2 ± 0.1b	4.3 ± 0.3c

a Different letters within each column indicate significant mean differences within each genotype according to Tukey’s test (*p* ≤ 0.05). All data are expressed as mean ± SD, *n* = 5.

Regarding nickel (Ni), an increase of the concentration of about 30% was detected in the Se-biofortified green pea microgreen with respect to the control. Some studies suggested that the application of exogenous amino acids, including histidine, glycine, and glutamine, can enhance the symplastic-to-apoplastic Ni ratio in root and promote the translocation of this metal to shoots. It is probable that Se ions or Se-bound amino acids may similarly contribute to the symplastic uptake and subsequent translocation of Ni, thereby enhancing the plant tolerance to Ni, but more research is required to substantiate this assertion.^34,38^ Other findings have reported the influence of Se on the accumulation and transport of Cu and Ni. The assimilation of Se by plants has been observed to modify the ionic permeability coefficient within the cell plasma membrane, consequently influencing the uptake of other ions such as micronutrients.^34,39^ Additionally, it is of great importance to mention that Ni is extremely important for nitrogen (N) metabolism in plants. In the case of leguminous plants, due to the nodulation process and the N fixation, there is a critical requirement of Ni.^40^ Therefore, it could be suggested that in green pea the concentration of Se applied acted as a biostimulant.

Among the plant species, alfalfa and, to a lesser extent, green pea showed greater levels of iron (Fe) and molybdenum (Mo) than red radish. Se enrichment did not alter the uptake and translocation of Mo and Fe, which is important since these elements play a key role in symbiotic N fixation by legumes. Red radish microgreens showed the greatest amount of P, Ca, Mg, and S with significant differences compared to the other plant species. Regarding S, this result is not surprising because cruciferous plants (red radish) have a greater requirement for S as they have abundant secondary metabolites of the glucosinolate type. These natural chemicals contribute to the plants’ defense against pests and diseases and give a characteristic bitter taste to Brassicaceae vegetables. It is also interesting to note that S shares similar properties with Se and a reduction in the uptake and accumulation of S and therefore a consequent substitution of S amino acids for Se amino acids could be expected with negative changes in the protein structure resulting in Se toxicity to plants.^41^ However, as already mentioned, no symptoms of toxicity were observed in the microgreens in terms of biomass, and, in addition, an increase in S accumulation was observed under Se biofortification in the cruciferous microgreen (red radish), which has a high nutritional requirement of this element. Table S1 also details the mineral concentrations present in tap water used for microgreen irrigation. The Se concentration existing in tap water was 0.81 ± 0.33 μg L^–1^

### 3.3 Effect of Se Biofortification on Bioactive Composition and Antioxidant Capacity

The results regarding chlorophyll and carotenoid concentrations are summarized in Table 2. Chla is always higher than Chlb in all of the samples as it is the primary photosynthetic pigment. Chlb is mainly produced from adaptation to shade to increase the light-harvesting process at low irradiances and it is not necessary for photosynthesis.^42^ The concentration of total Chls in Se-biofortified microgreens ranged from 205 to 579 mg·100 g^–1^ DW. The highest was obtained for alfalfa, followed by green pea, and the lowest was found in red radish. The level of carotenoids in Se-biofortified microgreens ranged from 58 to 119 mg·100 g^–1^ DW following the trend alfalfa > green pea > red radish. Greater values of total Chls, Chla, and carotenoids were observed in green pea enriched with Se, showing significant differences compared to the control. Previous studies demonstrated that Se application can increase the biosynthesis of photosynthetic pigments in plants, which has a protective effect on chloroplasts over the damage caused by ROS and environmental stress.^6^ Various studies reported that Se can control the photosynthesis antenna complex, defending chlorophylls by increasing the levels of photosynthetic pigments. It is possible that the advantages of Se in photosynthesis are related to the interaction of the Fe–S complex in chloroplasts since they play a crucial role in the electron transport chain and ensure that the high excitations of electronic levels have enough substrates to maintain the level of organization.^43^ Earlier reports demonstrated that the application of Se can encourage a restructuring of the antenna complex to increase the energy uptake and protect it from oxidative stress.^44^ No significant changes were found in all of the pigments analyzed in red radish biofortified with Se. However, total Chls, Chla, and Chlb decreased in Se-biofortified alfalfa, but carotenoid concentration was not affected. This suggests that the concentration of Se applied to alfalfa could have acted as a stress condition that would have affected the photosynthetic pigments, particularly chlorophylls. Notably, carotenoids, functioning as nonenzymatic antioxidants to counteract the formation of ROS by chloroplasts and peroxisomes, were not affected by Se due to the presence of selenate. Khan et al.^43^ stated that the presence of selenate could enhance the production of carotenoids, while the selenite form promotes the production of chlorophyll *b*. In this case, the joint effect of both forms of Se used could have altered the chlorophyll content but maintained the carotenoid levels in this type of plants. However, the observed Se effect, although it influenced the chlorophyll levels, maintained a homeostatic state without negatively affecting productivity. The lower levels of these pigments in Se-biofortified alfalfa could be correlated with the lower Mg concentration present compared to the control (see Table 1). Since Mg is the metal center of Chls molecules, it plays a vital role in Chls biosynthesis and in the activation of the plant photosystem.^45^ In a study performed on maize plants,^46^ it was also found that a reduction in Chls was associated with Mg deficiency in the plant, which corroborates the result obtained in our study. Hamilton^47^ identified three levels regarding the biological activity of Se. The first level proposes a low Se dose to promote plant growth, development, and enhanced beneficial effects. The second advocates for a moderate Se dose to support homeostatic processes, while the third level indicates that a high Se dose may lead to adverse consequences. According to these three levels, our results agree with those observations, green pea with the first level and red radish and alfalfa with the second one, thus suggesting that the plants were influenced by Se and had a different response depending on their tolerance to it.

**Table 2:** Bioactive Compounds (Chlorophylls, Carotenoids, and Total Phenolics) and Total Antioxidant Capacity (TAC) in Red Radish, Green Pea, and Alfalfa Microgreens Treated with Se and Their Respective Controls^a^

	chlorophyll *a* (mg 100 g^–1^)	chlorophyll *b* (mg 100 g^–1^)	total chlorophyll (mg 100 g^–1^)	carotenoids (mg 100 g^–1^)	TPC (mg GAE g^–1^ FW)	TAC (μmol Trolox g^–1^ FW)
red radish	145 ± 11a	35 ± 9a	177 ± 22a	55 ± 2a	1.9 ± 0.1a	9.5 ± 0.3a
red radish + Se	158 ± 17a	37 ± 9a	205 ± 36a	58 ± 2a	1.5 ± 0.2b	6.7 ± 0.7b
green pea	292 ± 63b	115 ± 8b	408 ± 66b	71 ± 26a	0.99 ± 0.04c	4 ± 1c
green pea + Se	404 ± 78c	118 ± 17b	522 ± 94c	116 ± 26b	0.9 ± 0.1ce	3.3 ± 0.5ce
alfalfa	560 ± 42d	156 ± 9c	719 ± 36d	148 ± 21b	0.70 ± 0.06d	2.4 ± 0.9de
alfalfa + Se	451 ± 16c	133 ± 15b	579 ± 10c	119 ± 16b	0.73 ± 0.07de	2.1 ± 0.7d

a Values are means ± SD of each plant species (*n* = 3). Tukey’s significance at *P* ≤ 0.05 among the treatments and plant species is indicated by different letters within each column.

The TPC in green pea and alfalfa did not show significant differences between the Se treatment and the control (Table 2); however, a 19% reduction in the TPC of red radish was found upon Se biofortification. Interestingly, the highest concentration of TPC in the Se-enriched genotypes was found in red radish with 1.5 ± 0.2 mg GAE·g^–1^ FW, followed by green pea and alfalfa with 0.9 ± 0.1 and 0.7 ± 0.07 mg GAE·g^–1^ FW, respectively. A decrease in phenolic compounds has also been reported in tomatoes biofortified with Se when concentrations greater than 25 μM Se were applied.^48^ D’Amato et al.^49^ observed a general increase in the levels of free and conjugated phenolic acids compared to the control when studying rice sprouts but found certain irregular variations that did not correlate with the expected level according to the Se concentration applied. In addition, the authors reported a decrease in the bound total phenolic acids upon Se biofortification. One potential elucidation for the diminished TPC observed in red radish Se-biofortified plants could be attributed to the plant surpassing its capacity to regulate Se tolerance. Consequently, this could have influenced the production of phenolic compounds, leading to reduced levels of these metabolites. Previous investigations have reported that when a plant surpasses its tolerance to Se, it can inactivate the antioxidant metabolism and glutathione depletion, which could evoke an altered cellular redox state and possible suppression of the phenolics biosynthesis.^50^

TAC analysis (Table 2) showed the same tendency found in TPC. As this analysis was carried out from the same methanolic extract used for TPC, it is not surprising to note a strong correlation between both methods. The highest equivalent antioxidant activity of Trolox in microgreens enriched with Se was found in red radish with 6.7 ± 0.7 μmol·g^–1^ FW, followed by green pea and alfalfa with 3.3 ± 0.5 and 2.3 ± 0.7 μmol·g^–1^ FW, respectively. These values correspond to a 29% decrease in radical scavenging activity in Se-treated red radish, but no significant differences were found between green pea, alfalfa, or their respective controls. It should be noted that the antioxidant activities of foods are highly dependent on the phenolic content, as well as other antioxidants present in food.^9^ In our study, they could be the main contributor to TAC and could also explain the similar trend found between both methods.

### 3.4 Glucose and Fructose Analyses

**Figure 2:**
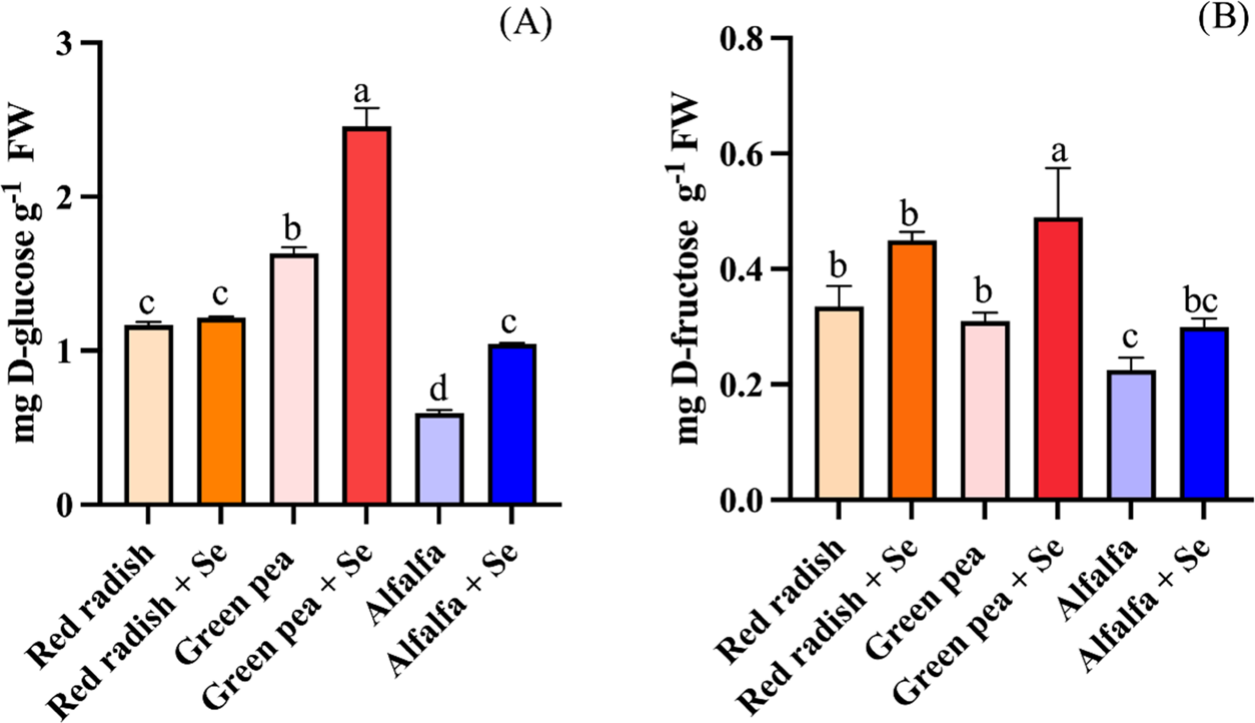
Concentration of d-glucose (A) and d-fructose (B) in red radish, green pea, and alfalfa microgreens treated with selenium and their respective controls. Means (±SD; *n* = 3) with different letters on top of the bars are significantly different (*p* < 0.05) according to Tukey’s test.

Overall, green pea had the greatest concentration of glucose and fructose followed by red radish and the lowest concentration found by alfalfa (Figure 2). The values ranged from 0.6 to 1.6 mg d-glucose g^–1^ FW and 0.2 to 0.3 mg d-fructose·g^–1^ FW. Similar concentrations were found in other microgreens with ranges between 0.2 and 4.7 mg glucose·g^–1^ FW and 0.8 and 5.6 mg fructose·g^–1^ FW.^51^ Under Se treatment, a significant increase of the glucose level of 51 and 76% was observed in the leguminous species, green pea, and alfalfa, while red radish showed no change (Figure 2A). Similarly, fructose was increased significantly in Se-biofortified green pea (58%), while in red radish and alfalfa, no statistically significant variations were found for this compound, but an increasing trend was observed in both (Figure 2B). Previous studies have shown that the application of Se could increase soluble sugar and regulate sugar metabolism. An increase of the level of Se can increase the activity of different enzymes involved in the regulation of the sugar metabolism of plants and the synthesis of sugars such as glucose or fructose.^52^ The high levels of sugars found when applying Se to microgreens in our research are consistent with previous findings in pea sprouts,^52^ alfalfa,^53^ red radish,^54^ and tomato plants biofortified with Se.^55^ We hypothesize that Se did not cause a significant effect on red radish, a cruciferous plant, due to the high amount of glucosinolates intrinsic to the plant (derived from glucose and amino acid).^56^ Kaur et al.^57^ indicated that the accumulation of Se can cause alterations in carbohydrate metabolism that depend on the concentration of Se, the ionic form of Se used in the application, the type of plant, and the stage of development of the plant. This could also explain the different trends and concentrations of these sugars found among microgreens.

### 3.5 Selenium Speciation Analysis

**Figure 3:**
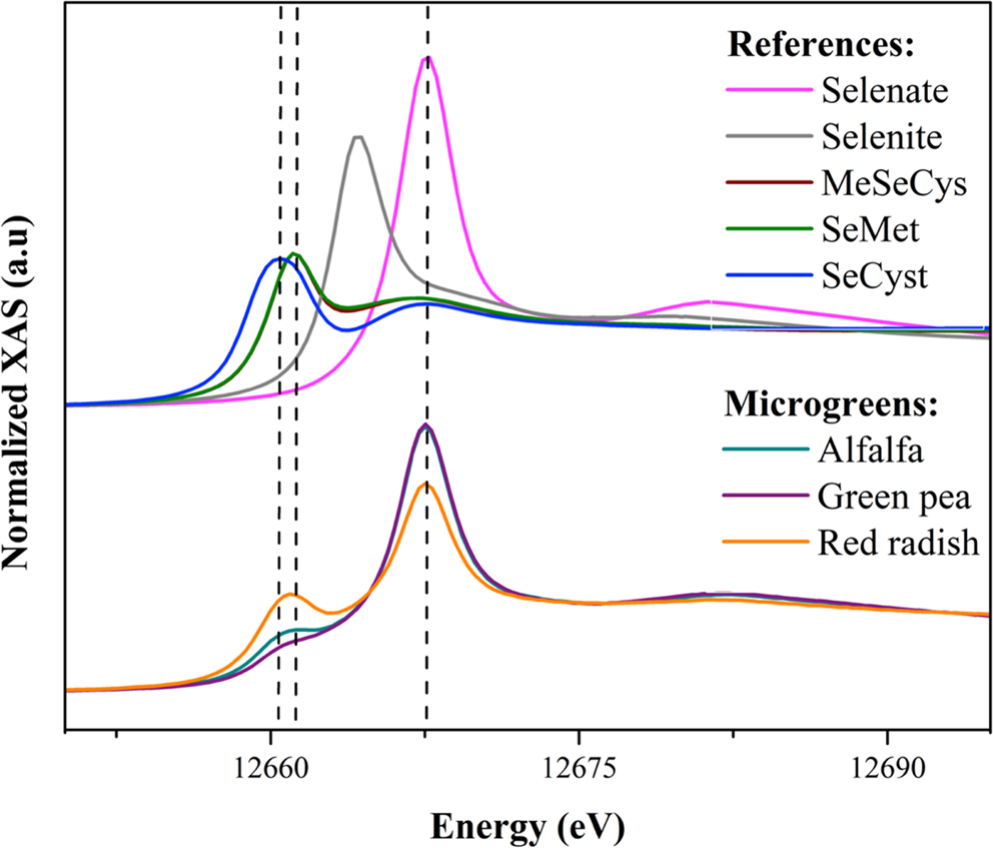
Normalized Se–K-edge XANES spectra of Se references (top) and microgreens Se-biofortified (bottom). The spectra have been shifted vertically for the sake of comparison.

The chemical speciation of Se in microgreens was determined using XANES spectroscopy. Figure 3 displays a comparison of the spectra collected on the Se references with those obtained from microgreens. Selenate and selenite species can be distinguished by their pronounced white-line (first resonance after the absorption edge). On the other side, SeMeCys and SeMet selenoamino acids have spectra alike as the Se atom has a similar coordination environment for both, C–Se–C. Hence, these compounds have been grouped as C–Se–C. Nevertheless, the spectral profile of SeCyst (C–Se–Se–C) significantly differs from those of C–Se–C. The *E*_0_ of these organic references appears at lower energies compared to the inorganic ones, and the white-line is significantly wider.^15^

By employing reference spectra, LCF analysis (Table S2) facilitated the determination of the species contributing to each sample spectrum. In red radish treated with Se, selenate (38.2 ± 0.2%) and C–Se–C species (37 ± 1%) were the dominant contributions followed by C–Se–Se–C species (22 ± 0.9%) and with a small amount of selenite species (2.6 ± 0.3%). Green pea Se-biofortified showed mainly the presence of selenate species (66 ± 0.3%) and a contribution of the organic species of C–Se–C (22 ± 2%) and low levels of selenite and C–Se–Se–C species (6.2 ± 0.5%; 6 ± 2%, respectively). Se-enriched alfalfa showed similar results to green pea, and predominantly selenate species and C–Se–C were found (62.2 ± 0.2 and 22 ± 1%, respectively) followed by C–Se–Se–C and selenite species (11.8 ± 1.2 and 4.3 ± 0.4%, respectively). According to these results, red radish accumulated more organic species compared to the others. Table S3 also shows that even though red radish and alfalfa accumulated similar concentrations of total Se, red radish biotransformed more Se into the organic forms to a higher degree compared to alfalfa and green pea. In the case of green pea, it is observed that despite being the microgreen with the most total Se accumulated, it is the plant with the lowest concentration of organic Se compounds. Our results are consistent with previous studies in wheat and alfalfa where it was found that, when biofortifying with a mixture of the inorganic species of Se, the main species present were the nontransformed selenate followed by C–Se–C species.^12,15^ The metabolism of Se in plant species varies among plants, meaning that different varieties can produce different Se chemical forms at various concentrations. Funes-Collado et al.^12^ reported that selenate was the major inorganic species (24%), and SeMet (15%) was the main organic species in alfalfa Se-biofortified with 25.3 μM of a mixture of selenate and selenite (1:1). However, they also accumulated large quantities of selenite (21%) and low levels of SeCyst (4.3%). Apart from the possible losses in efficiency due to the need for sample pretreatment in the indirect speciation performed in that study, those results suggest that the variations found can also be influenced by the different plant growth stages and the experimental conditions.

The high amount of the nontransformed selenate species found in the three microgreens could be explained by the fact that selenate is more easily transported to shoots than selenite or organic forms. The translocation process relies on various factors, including the xylem loading rate, plant transpiration, and physiological and environmental conditions,^41^ as well as the diffusion coefficient of the particular species. Selenate, for instance, exhibits an efficient movement from root epidermal cells to the xylem, resulting in higher Se concentrations in xylem exudates compared with selenite treatments. This mobility through the xylem is influenced by the species’ diffusion coefficient in solution. Selenate demonstrates a significantly higher diffusion coefficient, being 2–3 orders of magnitude greater than that of selenite in various media and conditions, whereas the diffusion coefficients of organic Se species fall in between these two extremes.^2,15^

Furthermore, the metabolic pathways of the Se species exhibit variability. Selenate undergoes reduction to selenite before subsequent conversion to selenide and, eventually, to organic species.^41^ There is a more rapid translocation of selenate to the aerial portions of the plant in comparison to its reduction, resulting in the preferential accumulation of selenate in shoots. A similar phenomenon is observed in the case of sulfur. Sulfate undergoes assimilation and reduction within the chloroplasts, but if the concentration in the xylem surpasses a certain threshold, it is also stored within the vacuoles of the leaf mesophyll cells. Sulfate residing in the vacuoles remains unmetabolized, exhibiting a benign impact on the plant and seldom being remobilized. Analogously, selenate follows a comparable pattern, accumulating without undergoing metabolism within the vacuoles of shoots, thereby eliciting no toxicity response.^15^ It is worth mentioning that selenate transportation across the cell membrane is an energy-dependent process mediated by the sulfate transport system.^2^ On the other hand, the low quantities of selenite in our results could be explained by the fact that this compound, which is absorbed by phosphate transporters and aquaporins (OsNIP2), is more concentrated in the root systems instead of being transported to the aerial parts due to the rapid transformation into organic forms of Se.^2,41^

### 3.6 Principal Component Analysis

**Figure 4:**
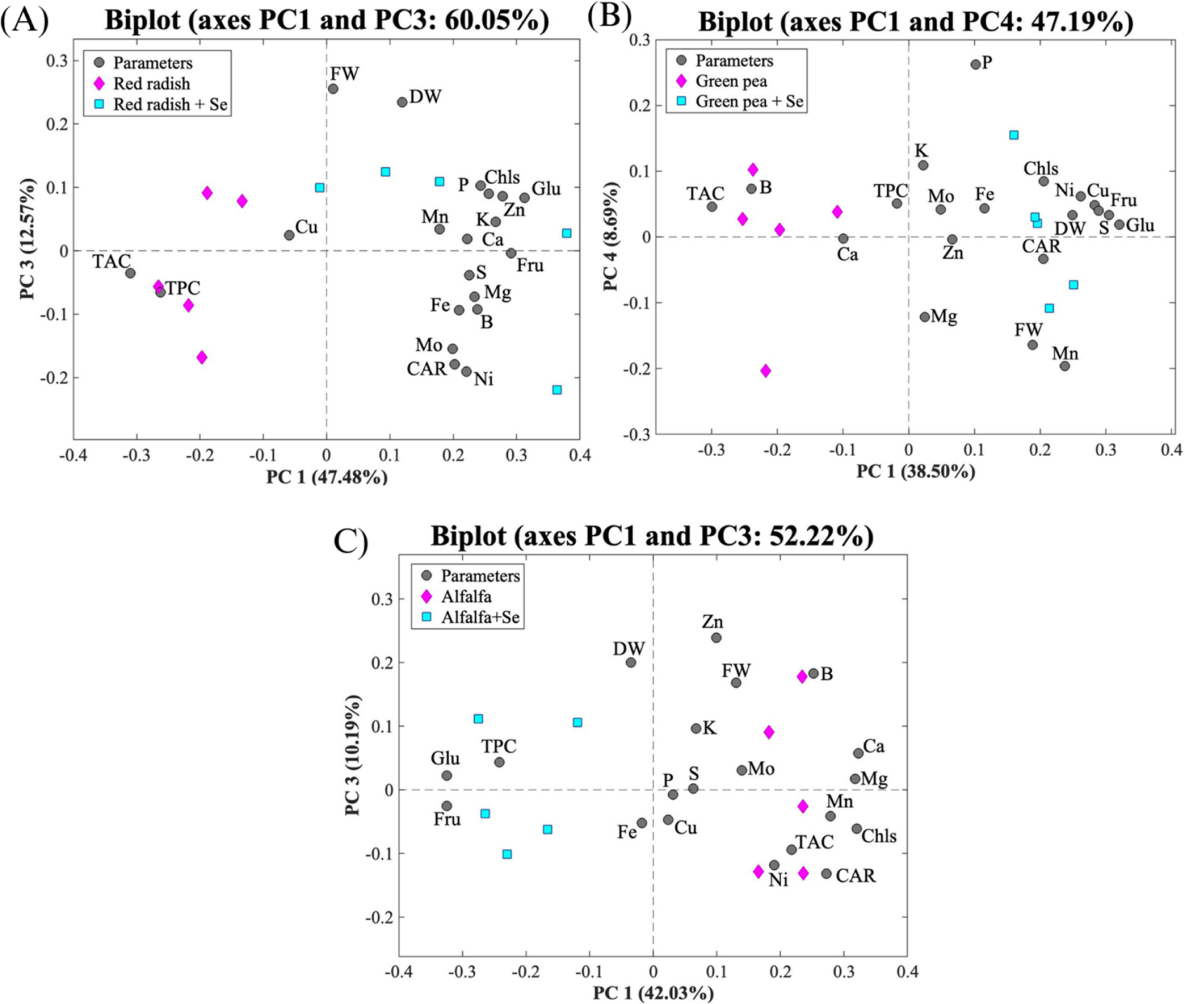
Principal component analysis of the different analyses performed on each microgreen treated (turquoise) and nontreated with Se (magenta): red radish (A), green pea (B), and alfalfa (C). The following parameters are included in the PCAs: TAC, TPC, Glu, Fru, Chls, Car, DW, FW, and macro- and micronutrients.

Principal component analysis (PCA) was performed to find correlations between Se application and the different measured parameters (mineral concentration, antioxidant activity, total phenolic compounds, sugars, pigments, and biomass) in each microgreen (Figure 4A–C). The proportion of the explained variance accumulated by the first and third components (PC1 and PC3) for red radish and alfalfa and the first and fourth components (PC1 and PC4) for green pea were approximately 60, 52, and 47%, respectively. As shown in Figure 4A, PC1 for red radish explained most of the total variation (48%) and it separated the Se-biofortified and the control in the positive and negative sides of PC1, respectively. PC3 explained 13% of the total variance. Selenium-biofortified red radish (positive PC1) is correlated with pigments (Chls and Car), FW, DW, sugar content (Glu and Fru), and macro- and microelements (Mg, P, Ca, S, K, Mn, B, Ni, Zn, and Mo), whereas the control showed a correlation with TPC, TAC, and Cu. Similarly, as seen in Figure 4B, PC1 of green pea explained most of the variance with 39%, and PC4 explained 9%. Se-biofortified green pea appears on the positive side of P1 and is positively correlated with pigments (Chls and Car), FW and DW, some macro- and microelements (Mg, P, S, K, Mn, Ni, Cu, Zn, Mo), and sugars (Glu and Fru). On the negative side of PC1 was located green pea non-Se-biofortified and was correlated mainly with TAC, B, Ca, and TPC. In Figure 4C, PC1 of alfalfa explained most of the total variation (42%) and separated the Se-biofortified and the control in the negative and positive sides of PC1, respectively. PC3 explained 10% of the total variance. Alfalfa control (positive PC1) was correlated with TAC, pigments (Chls and Car), FW, and macro- and micronutrients (Mg, P, S, K, Ca, Mn, B, Ni, Cu, Zn, and Mo), while Se-biofortified alfalfa (negative PC1) was correlated with TPC, Fe, DW, and sugars (Glu and Fru).

The PCA results highlighted the relevance of the Se biofortification effect on the different parameters measured in the microgreens studied. Thus, the different PCAs between the three microgreens suggest a greater impact of Se in more parameters to green pea and red radish than to alfalfa. This allowed us to have a comprehensive view of the correlations found between the bioactive compound and biological activity.

In the present study, it has been shown that the treatment of different species of microgreens with a mixture of selenite and selenate at a total concentration of 20 μM enhanced the Se content without reducing the yield, thus revealing the effectiveness of biofortification of microgreens with Se. Green pea was the microgreen that accumulated the highest Se concentration, followed by red radish and alfalfa. The levels of some essential nutrients in the studied microgreens were significantly reduced or increased by the application of Se. Nutrients such as K, P, S, and Mn were enhanced in Se-treated red radish, but Ca and Mg were reduced in alfalfa. Regarding phenolic compounds and antioxidant capacity, Se caused a significant decrease only in red radish, and no negative effect was observed in peas and alfalfa. Furthermore, Se treatment increased the concentration of soluble sugars (glucose and fructose) in green pea and alfalfa, but no significant changes were found in red radish. Thus, the positive effect of Se on carbohydrate metabolism is corroborated. Pigment concentrations (chlorophylls and carotenoids) increased with Se enrichment in green pea, and chlorophylls were slightly reduced in alfalfa. Our results point out that the effect of the Se mixture could have induced a decrease in the chlorophyll content while concurrently preserving the carotenoid levels in alfalfa. However, the observed Se effect, despite its influence on chlorophylls levels, sustained a homeostatic condition without impacting on the productivity. Regarding Se speciation, red radish was the microgreen that showed the highest level of selenoamino acids compared with the other two microgreens. Variation in organic Se concentrations, which depend on tolerance levels inherent to specific plant varieties, can be exploited to increase the nutritional benefits associated with the consumption of Se-biofortified microgreens as a functional dietary source. In conclusion, Se improved some parameters or reduced others depending on the plant species. Among the microgreens investigated, the Se-enriched green pea exhibited the most pronounced enhancements in acquired traits upon Se exposure, surpassing the other two microgreens. Conversely, red radish demonstrated a greater profile in organic Se compounds, which are known to be more bioavailable to humans. In any case, the three microgreens could be a good source not only of this essential nutrient for animals and humans but also of bioactive compounds such as carotenoids and chlorophylls that improve the nutritional profile of human health.

## Supporting Information Available

Mineral composition in tap water used for irrigation; Se species weight components resulting from linear combination fitting analysis over microgreens Se K-edge XANES spectra; and total Se inorganic and organic species accumulated according to Se total (100%) in each microgreen (PDF)

## Abbreviations

Se: selenium
SeCyst: selenocystine
SeMet: selenomethionine
SeCys: selenocysteine
SeMeCys: selenomethylcysteine
XAS: X-ray absorption spectroscopy
FW: fresh weight
DW: dry weight
Chls: chlorophylls
Car: carotenoids
TPCs: total phenolic compounds
TAC: total antioxidant capacity
GAEs: gallic acid equivalents
TEAC: Trolox equivalent antioxidant capacity
Glu: d-glucose
Fru: d-fructose
XANES: X-ray absorption near-edge structure
LCF: linear combination fitting
RDA: recommended daily Se allowance
