# Supplementary material for: Phytochemical Profile, Bioactive Properties, and Se Speciation of Se-Biofortified Red Radish (Raphanus sativus), Green Pea (Pisum sativum), and Alfalfa (Medicago sativa) Microgreens
Source: J Agric Food Chem. 2024 Feb 23;72(9):4947–57. doi: 10.1021/acs.jafc.3c08441 (PMC10921463; doi:10.1021/acs.jafc.3c08441)
Supplement: Supplementary file 1 — jf3c08441_si_001.pdf [file jf3c08441_si_001.pdf]

Supporting information

Phytochemical profile, bioactive properties, and Se speciation of Se-biofortified red radish (*Raphanus sativus*), green pea (*Pisum sativum*), and alfalfa (*Medicago sativa*) microgreens

Marilyn M. García-Tenesaca[1], Mercè Llugany[2], Roberto Boada[1*], María-Jesús Sánchez-Martín[1], Manuel Valiente[1]

[1] GTS-UAB Research Group, Department of Chemistry, Faculty of Science, Universitat Autònoma de Barcelona, 08193, Bellaterra, Spain

[2] Plant Physiology Group (BABVE), Faculty of Biosciences, Universitat Autònoma de Barcelona, 08193, Bellaterra, Spain

Table S1. Mineral composition in tap water used for microgreens irrigation.

**Macronutrients (mg L^{-1})**				
**Mg**	**P**	**S**	**K**	**Ca**
20.3± 0.6	0.31± 0.05	49.21 ± 0.03	14 ± 4	39 ±1

**Micronutrients (µg L^{-1})**							
**Mn**	**B**	**Fe**	**Ni**	**Cu**	**Zn**	**Mo**	**Se**
1.6± 0.9	119 ± 14	12 ± 4	6.2 ± 0.7	70 ± 16	0.06 ± 0.02	1.7± 0.9	0.8± 0.3

Values are means ± SD of each element (n = 3)

Table S2. Se species weight components resulting from linear combination fitting analysis of the Se K-edge XANES spectra collected over microgreens.

	**Relative concentration of species of selenium (%)**					
**Microgreens**	**Se^{4+}**	**Se^{6+}**	**C-Se-Se-C**	**C-Se-C**	**Reduced-χ2**	**R-factor**
**Red radish**	2.6 ± 0.3	38.2 ± 0.2	22 ± 0.9	37 ± 1	0.0002	0.001
**Green pea**	6.2 ± 0.5	66 ± 0.3	6 ± 2	22 ± 2	0.0007	0.002
**Alfalfa**	4.3 ± 0.4	62.1± 0.2	11.8 ± 1.2	22 ± 1	0.0004	0.001

R-factor is a measure of the mean square sum of the misfit at each data point which denotes the goodness of fit. The weight of each component is expressed as a percentage of the total. Values are means ± SD of each element (n = 3)

Table S3. Total Se inorganic and organic species accumulated according to Se total (100%) in each microgreen.

Microgreens	Se total (mg kg^{-1}DW)	Total Inorganic Se species (%)	Total Organic Se species (%)	Se inorganic in Total Se (mg kg^{-1}DW)	Se organic in Total Se (mg kg^{-1}DW)
**Red radish**	45 ± 9	41	59	18	27
**Green pea**	70 ± 16	72	28	50	20
**Alfalfa**	43 ± 13	66	34	28	15

Values of Se total are means ± SD of each element (n = 5)
